# Eating attitude and body satisfaction of adolescent and non-adolescent pregnant women

**DOI:** 10.1590/1806-9282.20241184

**Published:** 2025-03-31

**Authors:** Arife Soyturk, Nuray Egelioglu Cetisli

**Affiliations:** 1 Manisa City Hostipal – Manisa, Turkey.; 2 İzmir Kâtip Celebi University, Faculty of Health Sciences, Department of Nursing – İzmir, Turkey.

**Keywords:** Adolescent, Body satisfaction, Eating disorder, Nutrition

## Abstract

**OBJECTIVE:**

This study aimed to compare eating attitudes and body satisfaction between adolescent and nonadolescent pregnant women.

**METHODS:**

A descriptive and comparative study was conducted with 169 pregnant women, comprising 85 adolescents and 84 nonadolescents, who presented to the obstetrics outpatient clinic of a hospital in western Turkey. Data were collected using a Personal Information Form, the Multidimensional Body-Self Relations Questionnaire, and the Eating Attitudes Test-40. Descriptive statistics, Mann-Whitney U test, Kruskal-Wallis test, and Pearson correlation analysis were used for data analysis.

**RESULTS:**

The mean scores of the Eating Attitudes Test-40 were higher for adolescent pregnant women compared with nonadolescent pregnant women (p=0.040). It was determined that 15.3% of adolescent pregnant women and 4.8% of nonadolescent pregnant women were predisposed to eating disorders (p=0.038). The study found that the mean total score of the Multidimensional Body-Self Relations Questionnaire was lower for adolescent pregnant women compared with nonadolescent pregnant women, and the difference was statistically significant (p=0.000). Additionally, there was a statistically significant weak negative correlation between the scores of the Eating Attitudes Test-40 and the Multidimensional Body-Self Relations Questionnaire among adolescent pregnant women (r=-0.33, p<0.002).

**CONCLUSION:**

Adolescent pregnant women exhibit lower levels of body satisfaction and higher susceptibility to eating disorders compared with nonadolescent pregnant women.

## INTRODUCTION

The decision to have children is a pivotal moment in an individual's life. A woman's perception of pregnancy is influenced by various factors, including her physical and psychological health, quality of life, social relationships and environment, cultural and religious beliefs, and socioeconomic conditions^1^. The prevalence of eating disorders is higher among women of reproductive age compared with the general population^2,3^. During pregnancy, the recurrence of eating disorders is particularly notable, with a potential for these conditions to worsen^4^. Pregnancy is a period characterized by significant social, psychological, and physical changes in women. For adolescents, who make up approximately 20% of the global population, successful adaptation to pregnancy depends on maternal and fetal health concerns, as well as an emphasis on bodily and environmental awareness, which are crucial during adolescence^5,6^. The integration of pregnancy into adolescence, a critical transition phase from childhood to adulthood, can lead to serious health concerns and increased susceptibility to complications due to adolescents’ physical and mental unpreparedness for this process^7,8^.

During adolescence, a positive perception of body image and satisfaction can bolster self-confidence and enhance social interactions. In contrast, negative attitudes from family and peers can exacerbate body image distortion and body dissatisfaction^9^. The changes in dietary behaviors among adolescent pregnant women, coupled with their less healthful nutritional attitudes compared with adult pregnant women, can make their pregnancies more precarious^5,6^. The aspiration for thinness among adolescent women, alongside the psychological changes of this period, diverts them from healthy dietary habits and predisposes them to potential health complications^8,10^.

The experience and planning of pregnancy, role models within the adolescent's environment, social support (including partner support), and socioeconomic conditions collectively shape body image perception and influence decisions regarding the continuation and acceptance of pregnancy^11^. Adapting to the physical changes of adolescence can impede adolescents’ adjustment to and acceptance of pregnancy. According to a study, adolescent pregnant women tend to exhibit predominantly positive attitudes toward weight gain and body satisfaction, whereas those sensitive to weight gain and with eating disorders are more prone to negative health behaviors^12^. Sociocultural influences, particularly in societies with a thin-ideal body image promoted on social media, can lead to negative eating attitudes and body dissatisfaction among adolescents and pregnant women^11^. The further a pregnant woman strays from her body ideal, the more likely she is to experience negative eating attitudes and body dissatisfaction. Rapid body changes in adolescent pregnancies necessitate swift adaptation. Disparities in the physical appearance of an adolescent pregnant woman compared with her nonpregnant peers can exacerbate body dissatisfaction^12,13^. This dissatisfaction can lead to negative behaviors and perceptions of inadequacy. The concurrent experiences of adolescence and pregnancy can present significant challenges in terms of adaptation and acceptance^12^. Healthcare professionals should consider the body image perception of adolescent pregnant women to promote and protect maternal and infant health. It is crucial to be aware of the effects of public health interventions and health policies on this demographic^13,14^.

Health professionals have important responsibilities in preventing adolescent pregnancies, as well as in the healthy continuation and termination of pregnancies that occur. Body satisfaction, particularly critical in adolescents, along with the negative eating attitudes and behaviors developed to maintain this satisfaction, affects the pregnancy process, maternal–fetal health, childbirth, and the postpartum period^12^. Most literature reviews focus on the maternal and fetal outcomes associated with adolescent pregnancies. Information about eating disorders in adolescent pregnancies is quite limited in the literature. Therefore, there is a need for studies to elucidate the significance of this subject. This study aims to conduct a comparative analysis of eating attitudes and body satisfaction among pregnant adolescents and nonadolescent pregnant women.

## METHODS

The study employed a descriptive and comparative research design, targeting all pregnant women who visited the obstetric outpatient clinic of the hospital between October 2021 and May 2022. The sample size was calculated using GPower 3.1.9 software, assuming an effect size of 0.5, 80% statistical power, and a 0.05 margin of error, resulting in 64 pregnant adolescents and 64 nonadolescent pregnant women. Data were collected from 85 adolescent and 84 nonadolescent pregnant women who met the inclusion criteria. Post hoc analysis determined the study's power to be 90%.

The study included primiparous women beyond 28 weeks of gestation, without any psychological or physical illnesses that would prevent their participation, without pregnancy complications, aged 18–19 years for the adolescent group and over 20 years for the nonadolescent group, proficient in Turkish, and willing to participate voluntarily. Data were collected through face-to-face interviews and a questionnaire form in the clinic's consultation room, taking approximately 15–20 min on average.

The Personal Information Form examined the sociodemographic characteristics of pregnant women and consisted of 15 questions.

The Multidimensional Body-Self Relations Questionnaire (MBSRQ) was developed by Winstead and Cash^15^, with Turkish validity and reliability established by Doğan and Doğan in 1992, the MBSRQ assesses body image structure and individual attitudes. The 5-point Likert scale comprises 57 items across seven subdimensions: appearance evaluation, appearance orientation, physical competency evaluation, physical competency orientation, health evaluation, health orientation, and body areas satisfaction. Scores range from 57 to 285, with higher scores indicating a more positive body image. The Cronbach's alpha value was 0.94 in the Turkish study^16^ and 0.90 in this study.

Eating Attitudes Test-40 (EAT-40) was developed by Garner and Garfinkel^17^, the EAT-40 evaluates behaviors and attitudes associated with eating disorders and possible symptoms in individuals without eating disorders. The Turkish validity and reliability were established by Savasir and Erol in 1989. The six-point Likert scale comprises 40 items, with scores of 30 or above indicating a disposition toward eating disorders. The Cronbach's alpha value was 0.65 in the Turkish study^18^ and 0.77 in this study.

Data analysis was conducted using SPSS version 25.0. The sociodemographic characteristics of the participants are presented as number and percentage distributions. The Kolmogorov-Smirnov test assessed data normality, and since the data did not follow a normal distribution, nonparametric tests (Mann-Whitney U and Kruskal-Wallis tests) were used. Correlation analysis assessed the relationship between the MBSRQ and EAT-40 scores. A significance level of p<0.05 was used for all statistical analyses.

Approval was obtained from the Non-Interventional Clinical Research Ethics Committee (October 22, 2020, Decision No: 1016). Verbal and written consent was obtained from participants, adhering to institutional regulations. Permission to use the scales was obtained via email from the authors who conducted the Turkish validity and reliability studies.

## RESULTS

The sociodemographic characteristics of the participating mothers are presented in Table 1. Statistically significant differences were found between adolescent and nonadolescent pregnant women in terms of their mean age (t=-19.29, p=0.000), mean age of marriage (t=-17.177, p=0.000), education levels (χ^2^=39.724, p=0.000), employment status (χ^2^=15.580, p=0.000), and family types (χ^2^=20.451, p=0.000). The mean pre-pregnancy body mass index (BMI) of adolescent mothers was 22.75±4.48 kg/m^2^, while it was 24.23±5.00 kg/m^2^ for nonadolescent mothers, with the difference being statistically significant (t=-2.035, p=0.043). According to the Institute of Medicine (IOM) guidelines published in 2009 regarding gestational weight gain, 28.2% of adolescent mothers gained less weight than recommended, while 35.3% gained more weight than recommended. In contrast, 25% of nonadolescent mothers gained less weight than recommended, while 38.1% gained more weight than recommended. The incidence of unplanned pregnancy was more prevalent among adolescent mothers (31.8%), and this difference between the two groups was statistically significant (χ^2^=5.241, p=0.022) (Table 1). There was no statistically significant difference in the mean scores of the Eating Attitudes Test-40 and the Multidimensional Body-Self Relations Scale between adolescent and nonadolescent pregnant women based on their sociodemographic characteristics (p>0.05).

**Table 1:** The sociodemographic characteristics of pregnants.

Variables		Nonadolescent pregnants (n=84)		Adolescent pregnants (n=85)		Statistical analysis
		Mean±SD		Mean±SD		
Mean age (year) (min–max)		25.86±3.36 (22–38)		18.76±0.42 (18–19)		t=-19.291 p=0.000
Mean of marriage age (year) (min–max)		23.85±3.25 (18–35)		17.69±0.57 (16–19)		t=-17.177 p=0.000
Mean of gestational week (min–max)		37.73±2.60 (28–42)		38.38±1.66 (33–41)		t=1.938 p=0.054
Mean of pre-pregnancy body mass index (min–max)		24.23±5.00 (14.79–39.80)		22.75±4.48 (15.62–40.04)		t=-2.035 p=0.043
		**n**	**%**	**n**	**%**	
Education levels						
	Primary education and below	29	34.5	59	69.4	x^2^=39.724* p=0.000
	Secondary education	21	25.0	23	27.1	
	Higher education	34	40.5	3	3.5	
Employment status						
	Employee	29	34.5	8	9.4	x^2^=15.580 p=0.000
	Not employee	55	65.5	77	90.6	
Income status						
	Lower than expenses	4	4.7	3	3.5	x^2^=5.364* p=0.536
	Equal to expenses	54	64.3	68	80.0	
	Higher than expenses	26	31.0	14	16.5	
Family type						
	Nuclear family	77	91.7	53	62.4	x^2^=20.451 p=0.000
	Extended family	7	8.3	32	37.6	
Weight gained during pregnancy**						
	Low	21	25.0	24	28.2	x^2^=0.259 p=0.879
	Normal	31	36.9	31	36.5	
	High	32	38.1	30	35.3	
Planned pregnancy status						
	Planned	70	83.3	58	68.2	x^2^=5.241 p=0.022
	Unplanned	14	16.7	27	31.8	

* Fisher's exact test.

** The pre-pregnancy body mass index of the pregnant women was calculated and their BMI and weight gain according to their current gestational age were evaluated and classified according to the IOM 2009 guideline. SD: standard deviation.

In the study, the mean total score of the Eating Attitudes Test-40 for adolescent mothers was 20.35±8.99, with 15.3% found to have a predisposition to eating disorders. For nonadolescent mothers, the mean total score was 16.97±8.18, with 4.8% found to have a predisposition to eating disorders. A statistically significant difference was found between adolescent and nonadolescent mothers in terms of the mean total score of the Eating Attitudes Test-40 (U=2,770.50, p=0.040) and susceptibility to eating disorder predisposition (p=0.038). The mean total score of the Multidimensional Body-Self Relations Scale for adolescent mothers was 182.89±19.82, while it was 195.91±25.31 for nonadolescent mothers, and the difference between them was statistically significant (U=2,299.00, p=0.000). When examining the subscale mean scores of the Multidimensional Body-Self Relations Scale, it was found that adolescent mothers scored lower than nonadolescent mothers in the Appearance Evaluation (U=2,053.50, p=0.000),

Appearance Orientation (U=2,901.00, p=0.035), and Body Areas Satisfaction (U=2,312.50, p=0.000) subscales, with these differences being statistically significant (Table 2).

**Table 2:** The mean scores of the Eating Attitudes Test-40 and the Multidimensional Body-Self Relations Questionnaire of pregnants.

		Min–max	Nonadolescent pregnants (n=84)	Adolescent pregnants (n=85)	Statistical analysis
			Mean±SD	Mean±SD	
EAT-40 total score		0–120	16.97±8.18	20.35±8.99	U=2,770.00 p=0.040
Eating disorder disposition			**n (%)**	**n (%)**	
	Yes		4 (4.8)	13 (15.3)	p=0.038*
	No		80 (95.2)	72 (84.7)	
**MBSRQ total score**		57–240	195.91±25.31	182.89±19.82	U=2,299.00 p=0.000
Appearance evaluation		6–30	20.90±4.13	17.58±4.59	U=2,053.50 p=0.000
Appearance orientation		10–50	36.64±6.76	33.58±3.74	U=2,901.00 p=0.035
fitness evaluation		6–30	22.04±3.55	21.58±3.49	U=3,202.50 p=0.246
Fitness orientation		9–45	23.47±4.35	23.29±3.72	U=3,536.00 p=0.915
Health evaluation		6–30	21.09±3.75	20.35±4.11	U=3,217.50 p=0.266
Health orientation		11–55	40.60±5.68	39.74±5.43	U=3,284.50 p=0.368
Body areas satisfaction		9–45	31.14±7.11	26.74±6.60	U=2,312.50 p=0.000

* Fisher's exact test. SD: standard deviation; EAT-40: Eating Attitudes Test-40; MBSRQ: Multidimensional Body-Self Relations Questionnaire.

A statistically significant weak negative correlation was found between the total score of the Eating Attitudes Test-40 and the Multidimensional Body-Self Relations Scale among adolescent mothers (r=-0.331, p=0.002), as well as with the Appearance Orientation subscale (r=-0.358, p=0.001) and the Health Evaluation subscale (r=-0.334, p=0.002). No correlation was found between the total scores and subscale scores of the Eating Attitudes Test-40 and the Multidimensional Body-Self Relations Scale among non-adolescent pregnant women (p>0.05) (Table 3).

**Table 3:** The correlation between pregnants of Eating Attitudes Test-40 and the Multidimensional Body-Self Relations Questionnaire mean scores.

		EAT-40	MBSRQ	Appearance evaluation	Appearance orientation	Fitness evaluation	Fitness orientation	Health evaluation	Health orientation	Body areas satisfaction
Nonadolescent pregnants	EAT-40									
	MBSRQ	r=-0.081 p=0.466								
	Appearance evaluation	r=-0.147 p=0.182	r=0.678 p=0.000							
	Appearance orientation	r=-0.037 p=0.736	r=0.750 p=0.000	r=0.371 p=0.001						
	Fitness evaluation	r=-0.044 p=0.690	r=0.846 p=0.000	r=0.600 p=0.000	r=0.691 p=0.000					
	Fitness orientation	r=0.016 p=0.886	r=0.518 p=0.000	r=0.001 p=0.996	r=0.308 p=0.004	r=0.340 p=0.002				
	Health evaluation	r=-0.145 p=0.187	r=0.590 p=0.000	r=0.227 p=0.038	r=0.378 p=0.000	r=0.557 p=0.000	r=0.442 p=0.000			
	Health orientation	r=0.088 p=0.425	r=0.808 p=0.000	r=0.466 p=0.000	r=0.506 p=0.000	r=0.613 p=0.000	r=0.491 p=0.000	r=0.309 p=0.004		
	Body areas satisfaction	r=-0.1471 p=0.181	r=0.753 p=0.000	r=0.686 p=0.000	r=0.364 p=0.001	r=0.511 p=0.000	r=0.142 p=0.198	r=0.285 p=0.009	r=0.552 p=0.000	
Adolescent pregnants	EAT-40									
	MBSRQ	r=-0.331 p=0.002								
	Appearance evaluation	r=-0.055 p=0.620	r=0.506 p=0.000							
	Appearance orientation	r=-0.358 p=0.001	r=0.433 p=0.000	r=0.029 p=0.795						
	Fitness evaluation	r=-0.160 p=0.144	r=0.654 p=0.000	r=0.315 p=0.003	r=0.032 p=0.773					
	Fitness orientation	r=0.098 p=0.373	r=0.402 p=0.000	r=-0.143 p=0.193	r=0.008 p=0.941	r=0.196 p=0.072				
	Health evaluation	r=-0.334 p=0.002	r=0.536 p=0.000	r=0.122 p=0.264	r=0.081 p=0.460	r=0.492 p=0.000	r=0.216 p=0.047			
	Health orientation	r=-0.170 p=0.121	r=0.731 p=0.000	r=0.292 p=0.007	r=0.139 p=0.205	r=0.531 p=0.000	r=0.304 p=0.005	r=0.252 p=0.020		
	Body areas satisfaction	r=-0.104 p=0.341	r=0.567 p=0.000	r=0.382 p=0.000	r=-0.230 p=0.034	r=0.319 p=0.003	r=0.242 p=0.025	r=0.204 p=0.061	r=0.376 p=0.000	

EAT-40: Eating Attitudes Test-40; MBSRQ: Multidimensional Body-Self Relations Questionnaire.

## DISCUSSION

In the study, it was found that the mean score of the Eating Attitudes Test-40 for adolescent mothers (20.35±8.99) was higher compared with nonadolescent mothers (16.97±8.18), and susceptibility to eating behavior disorders was observed in 15.3% of adolescent mothers and 4.8% of nonadolescent mothers. The number of studies examining the dietary behaviors of adolescent mothers in the literature is quite limited. Similar to the findings of the current study, Mridha et al. reported that adolescent mothers experienced more nutrition-related problems compared with other pregnant women, with 60% having low dietary quality, 36% having low BMI, and 28% being anemic^19^. In the study conducted by Sarmiento and colleagues, it was found that 31% of adolescent pregnant women were underweight according to their BMI, and a higher incidence of iron deficiency and anemia was observed in adolescent pregnant women compared with nonadolescent pregnant women^20^. In a study evaluating the eating attitudes and body satisfaction of adolescent pregnant women in Brazil, it was determined that 11.9% of adolescent pregnant women consumed insufficient food, and 28.4% skipped meals^12^. Based on the findings obtained from the study and existing literature, it is believed that adolescent pregnant women tend to maintain their eating behaviors from pre-pregnancy during their pregnancies. Additionally, their priorities in nutrition are thought to lean toward preserving their own body image rather than prioritizing maternal and fetal health. In today's conditions, the acceptance of a thin appearance among adolescents also leads them to maintain this perception in their pregnancies. Research has found that adolescent pregnant individuals included in the study have lower body satisfaction compared to nonadolescent pregnant individuals.

In adolescent pregnancies, women are considered a high-risk group because they have to cope with all the positive and negative aspects of both adolescence and the pregnancy process. The bodily changes that occur during this period significantly affect the adolescent and their body satisfaction^12^. In a systematic review conducted by Zaltzman et al., which included six studies evaluating body image in adolescent pregnancies, some research reported an increase in body image disturbance and dissatisfaction during pregnancy among adolescents, while others reported that adolescent pregnant individuals had positive attitudes toward weight gain and body satisfaction^10^. In our country, a study conducted by Çırak aimed at evaluating the body image of adolescent pregnant individuals (n=165) found that adolescents were satisfied with their bodies^13^. Negative perception of body image during pregnancy or dissatisfaction with one's body can lead to negative attitudes and behaviors toward the pregnancy itself^12^. It is important for pregnant women to be evaluated holistically with their environment during the prenatal period, to increase their awareness of potential problems that may arise during pregnancy, and for nurses to provide education and counseling to strengthen their ability to cope with these issues. Positive body satisfaction will enable the adolescent pregnant individual to experience both the pregnancy and postpartum periods more healthily.

In the study, it was determined that pregnant adolescents who were susceptible to eating behavior disorders had a negative body image, while there was no correlation between these two variables in nonadolescent mothers. Similarly, in the study by Oliboni and Alvarenga, it was found that adolescent pregnant individuals generally exhibited positive attitudes toward weight gain and body satisfaction, whereas obese adolescent pregnant individuals experienced higher levels of body dissatisfaction and displayed negative eating behaviors^12^. Body dissatisfaction in adolescent girls can lead to unhealthy eating behaviors, unhealthy weight loss choices, and eating disorders such as anorexia nervosa and bulimia nervosa^8,10^. As dissatisfaction with body image perception increases among those with a negative body image, the likelihood of resorting to unhealthy methods increases, potentially leading to dangerous outcomes. Adolescent pregnant individuals who continue similar behaviors during their pregnancies may exhibit behaviors such as restriction of food intake, skipping meals, and altering their dietary patterns by avoiding essential nutrients like carbohydrates and proteins in pursuit of a thinner body image^21^. It is thought that the statistical difference in the pre-pregnancy BMI average of adolescent and nonadolescent pregnant women is a result that supports this idea.

This study had some limitations. The number of studies in the literature examining the relationship between body satisfaction and eating disorders in adolescent pregnant women was quite limited. This situation limited the discussion of the data obtained from the study in the context of literature. However, the most important strength of the study is that it is the first study conducted in this field and in our country.

## CONCLUSION

According to the findings of the study, it is recommended to conduct detailed evaluations of all adolescent pregnant individuals based on their sociodemographic and obstetric characteristics. Collaboration among various professional groups is crucial for creating guidelines aimed at fostering positive eating attitudes and body image among adolescent pregnant individuals, increasing awareness, and providing realistic professional psychological support for those with negative body image perceptions. Adolescents demonstrating negative eating attitudes should receive support and counseling from nutrition specialists. Education highlighting the importance of managing negative eating behaviors to prevent obstetric and fetal complications should be provided to adolescent pregnant individuals. Furthermore, nurses in primary healthcare settings, who spend significant time with pregnant women, should receive training to provide nutritional counseling specifically tailored for adolescent pregnant women. Comprehensive evaluation of adolescent pregnant individuals should assess their sociological, physiological, and psychological readiness for pregnancy. It is recommended to conduct broader studies with larger samples comparing eating attitudes and body satisfaction in adolescent pregnant individuals, and exploring factors influencing these conditions.

## Data Availability

Data will be made available upon reasonable request.
